# The Effects of Need Satisfaction and Dissatisfaction on Flourishing among Young Chinese Gamers: The Mediating Role of Internet Gaming Disorder

**DOI:** 10.3390/ijerph16224367

**Published:** 2019-11-08

**Authors:** Bryant Pui Hung Hui, Anise M. S. Wu, Nicolson Y. F. Siu, Ming-Lun Chung, Ngai Pun

**Affiliations:** 1 Department of Sociology, Faculty of Social Sciences, University of Hong Kong, Hong Kong, China; s898000335@gmail.com (M.-L.C.); npun@hku.hk (N.P.); 2 Department of Psychology, Faculty of Social Sciences, University of Macau, Avenida da Universidade, Taipa, Macao, China; anisewu@um.edu.mo; 3 Department of Counselling & Psychology, Hong Kong Shue Yan University, Hong Kong, China; yfsiu@hksyu.edu

**Keywords:** Internet gaming disorder, self-determination theory, psychological needs, flourishing, Chinese

## Abstract

Given the increasing popularity of online game playing, the negative impacts of game addiction on both adolescents and adults attracted our attention. Previous studies based on the self-determination theory have examined the effects of the three basic psychological needs of autonomy, competence, and relatedness on problematic video game playing among Chinese young adults. Yet, as more evidence emerged pointing to the possible relation between need dissatisfaction and higher vulnerability for ill-being and psychopathology, the present study aimed to incorporate the impacts of both satisfaction and dissatisfaction for autonomy, competence, and relatedness in explaining Internet gaming disorder (IGD), a condition that may in turn impede eudaimonic well-being as indicated by flourishing. In a self-administered online survey with a valid sample of 1200 Chinese young adults aged 18–24 years (mean age = 19.48 years), the prevalence of probable IGD (for those who reported five or more symptoms in the Diagnostic and Statistical Manual of Mental Disorders (DSM-5) symptom list for IGD) was 7.5%. Our results showed that relatedness dissatisfaction positively predicted IGD symptoms after controlling for other need satisfaction and dissatisfaction. Also, flourishing was found to be negatively predicted by IGD. Finally, IGD was found to mediate the effect of relatedness dissatisfaction on flourishing. Our findings suggested a risk factor of relatedness dissatisfaction in predicting IGD, thereby significantly predicting flourishing.

## 1. Introduction

Self-determination theory (SDT) postulates that the existence of three basic psychological needs—namely, autonomy (the need to experience choice and psychological freedom), competence (the need to feel effective or a sense of mastery), and relatedness (the need to feel connected with significant others)—is essential for people to develop their fullest potential and attain personal growth [1,2]. SDT is such a versatile theory that it has been widely applied in many research domains. There is ample scientific evidence suggesting that people who have met those psychological needs may experience high levels of personal well-being [3,4] and low levels of psychopathology, such as Internet gaming disorder (IGD) [5]. In a recent development of SDT, Sheldon and Gunz proposed a new construct of need frustration or dissatisfaction, which is related to, but distinct from, need satisfaction [6]. They provided empirical evidence that the unmet three psychological needs aroused corresponding desires to obtain the missing experiences, while a surfeit of need satisfaction did not predict a decrease in desires for the respective needs. A Balanced Measure of Psychological Needs, measuring both need satisfaction and need dissatisfaction, has been developed [7] and used for scientific research [8,9]. Benefiting from the theoretical and measurement advances in psychological need satisfaction and dissatisfaction, this study examined the independent effects of need satisfaction and dissatisfaction on IGD and flourishing.

Online gaming is a global concern [10], especially in China. According to the China Internet Network Information Center [11], the number of domestic online gamers has increased from 336 million to 422 million in the past five years, representing a growth rate of 25%. While recognizing the recreational function of online gaming [12,13], excessive and prolonged online gaming is shown to be associated with detrimental effects, including poor academic performance [14], low well-being, and high loneliness [15], as well as lack of real-life relationships [16]. More seriously and specifically, online gamers may eventually develop IGD, which has been labeled as a condition for further investigation in the Diagnostic and Statistical Manual of Mental Disorders (DSM-5) [17] and recognized as a new mental health condition in the World Health Organization’s 11th edition of International Classification of Diseases (ICD-11) [18]. Given that students from high schools, technical secondary schools, technical schools, and junior colleges accounted for a majority (55%) of Chinese online gamers [19], more research attention should be devoted to reducing IGD risks on campus.

Considerable research has been conducted on the relationship between psychological needs and gaming. For instance, a study done by Przybylski and colleagues has revealed that overall need satisfaction was negatively associated with obsessive passion, amount of play, and tension following play [20]. In particular, Wu and colleagues found in a Chinese adult sample that relatedness satisfaction negatively predicted problematic video game playing, while autonomy and competence satisfaction only showed indirect effects on problematic video game playing via purpose in life [5]. Meanwhile, some researchers attempted to enrich gaming studies by employing the latest development of need frustration or dissatisfaction in the field of SDT. For instance, a recent study on both need frustration and need satisfaction found that problematic gaming was positively correlated with need frustration and negatively correlated with need satisfaction in an online sample [21]. In a Taiwanese adolescent sample, Wan and Chiou found that the compulsive use of online games may be driven by the relief of need dissatisfaction, rather than the pursuit of satisfaction [22]. Other research only covered daily need frustration in their studies, and demonstrated how it could explain the increasingly problematic style of video gaming engagement in North American samples [23,24]. Collectively, previous research has suggested that psychological needs, especially when being dissatisfied, may predict IGD. However, to the best of our knowledge, as the dearth of empirical studies mentioned above illustrates, there are few studies that explicitly investigate the needs for autonomy, competence, and relatedness separately—except [5]—and the unique impact of satisfaction and dissatisfaction for the three needs by including them all in the same model.

While need dissatisfaction may evoke ill-being and higher vulnerability for psychopathology alluding to IGD, need satisfaction may foster well-being [25,26]. Two approaches can be adopted to understand well-being, namely the hedonic perspective and eudaimonic perspective [27]. In general, the hedonic perspective defines well-being as pleasure attainment and pain avoidance [28]. In contrast, the eudaimonic perspective holds that well-being is more than just pleasure—instead, this perspective is concerned with actualizing human potential [29]. Eudaimonic well-being is a process of realizing one’s daimon, or true self, which means acting congruously with one’s deeply held values and fulfilling one’s virtuous potentials [27,29]. Previous empirical studies on psychological needs have been mainly focusing on hedonic well-being. For instance, a research study sampling Chinese adolescent and college student participants found that need satisfaction composite positively predicted life satisfaction and positive affect, while negatively predicting negative affect [30]. Moreover, Sheldon and Elliot showed that need satisfaction significantly predicted subjective well-being both at the same time point and one semester later [31]. In spite of these aforementioned studies on hedonic well-being, there are few empirical studies explicitly examining psychological needs and eudaimonic well-being. The closest empirical clue only reveals that satisfaction of the three separate needs is positively associated with the two proxies for eudaimonic well-being, namely, self-esteem and subjective vitality [3,32]. More SDT research on eudaimonic well-being should be conducted to fill this research gap.

One of the latest indicators commonly used by researchers to evaluate eudaimonic well-being is flourishing [33,34]. Flourishing refers to optimal human functioning, and its core components include meaning and purpose, supportive and rewarding relationships, engagement and interest, contribution to the well-being of others, competence, self-acceptance, optimism, and being respected [35], though some argued it should be a combination of feeling good and functioning effectively [36]. A brief eight-item Flourishing Scale was developed to assess major aspects of a person’s social-psychological functioning [37], thereby creating a broad overview of the person’s psychological well-being. Similar to IGD research, another limitation of past SDT research on well-being is that most studies have only focused on need satisfaction as a whole or separately. Although some recent theoretical developments suggested that need satisfaction and dissatisfaction may have different effects on outcome variables [7], to the best of our knowledge, no studies have examined their potentially distinct effects on flourishing, being the understudied indicator of eudaimonic well-being in the existing literature.

In light of the above, and drawing from previous SDT research on IGD and well-being, we suggested that both IGD and flourishing can be examined in an SDT framework. Particularly, we studied how psychological need satisfaction and dissatisfaction predict IGD and flourishing. As a popular recreational activity, the major function of online gaming is personal entertainment and enjoyment cross-culturally [12,13]. However, when online gaming becomes excessive and pathological, the damage is likely to be catastrophic. Longitudinal evidence has consistently found problematic Internet use, including gaming, a prospective risk factor of ill-being (e.g., depression, anxiety, and social phobia) among youth [38,39,40]. Further research is warranted to examine the effect of Internet-related disorders on well-being, particularly the eudaimonic one.

Therefore, in the context of SDT, we examined (1) under what psychological need condition/s online gaming would develop into IGD and diminish/facilitate flourishing, and (2) how IGD would predict flourishing under such condition/s. In other words, the present study would contribute to our knowledge of how different psychological need conditions are related to the development of IGD. By incorporating the impact of need satisfaction and dissatisfaction for autonomy, competence, and relatedness in explaining IGD, it would further enhance our understanding of their relationships. In addition, through exploring flourishing, an indicator for eudaimonic well-being, it is expected to provide a more comprehensive understanding of different factors in predicting eudaimonic well-being.

## 2. Materials and Methods

### 2.1. Participants and Procedures

In late October, 2017, we invited all students (approximate *N* = 3000) in a higher education vocational college in Xi’an, China to take part in a self-administered online survey, which is part of a large-scale research project investigating the process of “learning-to-labor” among vocational college youth in China [41,42]. A total of 1649 respondents voluntarily participated in and completed the survey without any monetary incentives (response rate = 55%). To identify careless responses and ensure a relative high-quality dataset [43,44], six quality-check items (e.g., “For this item, please select ‘*strongly disagree*’”) were embedded in the survey. We only included those who responded according to the instruction to four or more quality-check items and finished the survey in 15 min or more, resulting in a final sample of 1200 respondents (826 males, 68.80%; *M*_age_ = 19.48, *SD*_age_ = 1.21, age range = 18–24 years). Online informed consent was obtained from all respondents, and this study was approved by the ethics committee of the affiliated university of the last author. The data collection was approved and assisted by the vocational college administration.

### 2.2. Measures

**Internet gaming disorder (IGD)**. IGD symptoms were measured with the nine diagnostic criteria for IGD listed in the DSM-5 [17]. These criteria have been used in previous research for the same purpose [13,42]. The respondents were asked whether each of the nine symptoms (e.g., unsuccessful attempts to control game involvement) could describe themselves in the past 12 months (0 = *No*, 1 = *Yes*). The internal consistency (KR-20) for this sample was 0.84. A higher summated score of these nine items represents a higher level of IGD tendency. We used a cut-off of 4/5 to identify probable IGD cases [45], and over 7.50 percent of the cases were classified as probable IGD.

**Basic psychological needs.** Psychological needs were assessed by a Chinese version of the 18-item Balanced Measure of Psychological Needs (BMPN) scale [7]. The items were translated and back-translated by separate Chinese–English bilinguals, and inconsistencies were resolved in a final discussion of the translation. Respondents were instructed to use a five-point Likert scale ranging from 1 (*no agreement*) to 3 (*some agreement*) to 5 (*much agreement*) to rate each statement based on their last two weeks. Under each of the six subscales, there were three items tapping the satisfaction and dissatisfaction of the three needs, namely autonomy satisfaction (e.g., I was free to do things my own way), competence satisfaction (e.g., I did well even at the hard things), relatedness satisfaction (e.g., I felt close and connected with other people who are important to me), autonomy dissatisfaction (e.g., I had to do things against my will), competence dissatisfaction (e.g., I struggled doing something I should be good at), and relatedness dissatisfaction (e.g., I had disagreements or conflicts with people I usually get along with). The Cronbach’s alpha α = 0.66, 0.80, 0.77, 0.42, 0.63, and 0.61, respectively. Due to the modest reliability of autonomy dissatisfaction, its relevant results should be interpreted with caution.

**Flourishing.** We used the validated Chinese version [34] of the eight-item Flourishing Scale [35], which is designed to measure important aspects of the aforementioned human functioning [37]. Participants rated each item on a seven-point Likert scale ranging from 1 (*strongly agree*) to 7 (*strongly disagree*). Sample items include: “I lead a purposeful and meaningful life” and “My social relationships are supportive and rewarding”. The Cronbach’s alpha α = 0.94.

**Background variables.** The questionnaire also consisted of demographic information, including respondents’ age, sex, and hukou (i.e., official household registration being rural or nonrural), which is a proxy for socioeconomic status [41]. Regarding daily gaming frequency, respondents were asked questions such as “On average how much time do you spend on gaming every day?”, and they had to choose a response from eight categories, ranging from less than one hour to seven hours or more.

### 2.3. Data Analysis

All statistical analyses were conducted with SPSS 25.0 (IBM Corp., Armonk, N.Y., USA). We first examined the descriptive statistics and bivariate correlations among all variables before testing our hypothesized model. A mediation analysis was then conducted to test whether the relationship between psychological needs and flourishing would be mediated by IGD. We employed PROCESS macro in SPSS (Model 4), which allowed us to simultaneously estimate the indirect effects of multiple predictors—through the same meditator—in a single model [46]. Bootstrapped mediation tests were conducted based on 5000 bootstrapped resamples.

## 3. Results

### 3.1. Descriptive Statistics

Table 1 shows the descriptive statistics of our variables of interest. To explore the statistical features of the variables, we calculated the mean and standard deviation of each variable, their reliability coefficients, and the Pearson correlation coefficients for each pair of variables. The correlational results showed that dissatisfaction, but not satisfaction, of autonomy, *r*(1198) = 0.13, *p* < 0.001, competence, *r*(1198) = 0.10, *p* = 0.001, and relatedness, *r*(1198) = 0.19, *p* < 0.001, were positively correlated with IGD. Flourishing was negatively correlated with IGD, *r*(1198) = −0.14, *p* < 0.001, autonomy dissatisfaction, *r*(1198) = −0.08, *p* = 0.008, and relatedness dissatisfaction, *r*(1198) = −0.22, *p* < 0.001—not competence dissatisfaction—while positively correlated with autonomy satisfaction, *r*(1198) = 0.43, *p* < 0.001, competence satisfaction, *r*(1198) = 0.51, *p* < 0.001, and relatedness satisfaction, *r*(1198) = 0.43, *p* < 0.001. These results were basically in line with our hypotheses, and thus we moved on to test them in a mediation model.

### 3.2. Mediation Analysis

To account for the shared association among predictors in the mediation model being estimated, the six psychological need variables (i.e., autonomy satisfaction, autonomy dissatisfaction, competence satisfaction, competence dissatisfaction, relatedness satisfaction, and relatedness dissatisfaction) are included in the models for both IGD (mediator) and flourishing. Furthermore, to prevent possible confounding and epiphenomenal association due to covariates, the four covariates of sex, age, hukou, and daily gaming frequency were included in the models.

**Figure 1:**
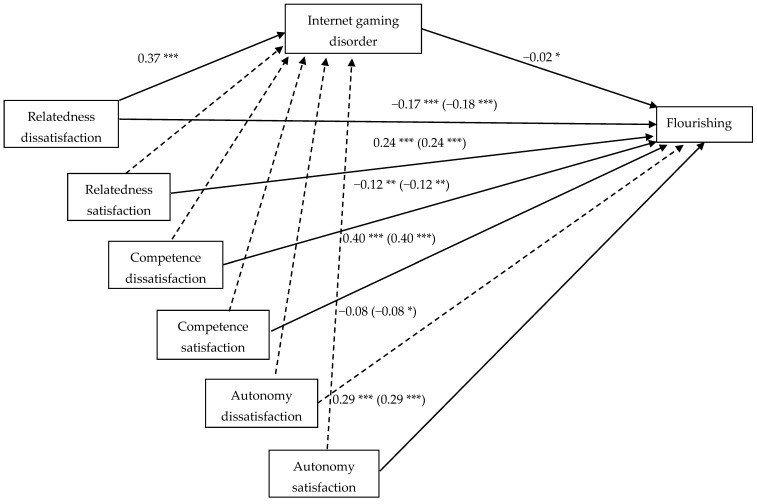
Unstandardized coefficients of a mediation model (controlling for sex, age, hukou, and daily gaming frequency) depicting Internet gaming disorder as a mediator between psychological needs and flourishing. *Note. N =* 1200. Coefficients in parentheses illustrate the total effect without the mediator. Dashed lines represent a nonsignificant relationship (*p* > 0.05). * *p* < 0.05. ** *p* < 0.01. *** *p* < 0.001.

All model coefficients are summarized in Table 2. In model 1, we first performed a regression analysis for the mediator model, which revealed that the paths from six psychological need variables to IGD were not significant, except for the predictor of relatedness dissatisfaction, *B* = 0.37, *p* < 0.001. Next, we adopted model 2 to test the total effect of psychological need satisfaction and dissatisfaction on the outcome variable of flourishing (i.e., without the mediator of IGD). The results showed that flourishing was significantly predicted by autonomy satisfaction, *B* = 0.29, *p* < 0.001, autonomy dissatisfaction, *B* = −0.08, *p* = 0.035, competence satisfaction, *B* = 0.40, *p* < 0.001, competence dissatisfaction, *B* = −0.12, *p* = 0.002, relatedness satisfaction, *B* = 0.24, *p* < 0.001, and relatedness dissatisfaction, *B* = −0.18, *p* < 0.001. Further, our model 3 showed that, in the presence of IGD as the mediator, the six psychological need variables—except for autonomy dissatisfaction—significantly predicted flourishing. Most importantly, the mediator of IGD also significantly and negatively predicted flourishing, *B* = −0.02, *p* = 0.010. Thus, we moved on to look into the indirect effects of the six psychological needs on flourishing through IGD. Bootstrapping results revealed that only the indirect effect of relatedness dissatisfaction on flourishing through IGD was significant, *B* = −0.009, with a 95% confidence interval (CI) excluding zero [−0.019, −0.002]. Figure 1 depicts the complete model corresponding to the paths of mediator, direct effect, and total effect in the form of a statistical diagram.

To protect our results from potential spurious associations between key variables caused by the covariates in our models, the outcomes including and excluding the covariates of sex, age, hukou, and daily gaming frequency were compared. It is shown that such covariates did not have substantive effects on the results.

## 4. Discussion

Owing to the increasing penetration of broadband Internet and mobile devices, including smartphones and tablets, in daily lives, IGD has become a worsening issue around the globe and China is no exception. Games designed with immersive or interactive features can be extremely engaging and require so much time and effort that they affect gamers’ daily routines [47,48]. Thus, the strong correlations between IGD and real-life problems found in previous studies warrant our attention [49,50]. Setting the cut-off of 4/5 for the nine-item DSM-5 diagnostic criteria [45], we estimated the prevalence of probable IGD to be 7.5% in our Chinese young adult sample. Although the present study did not adopt probability sampling, the prevalence of IGD is similar to other studies in the country. A systematic review found that the prevalence of problematic online gaming ranged from 3.5% to 17.0% [51]. Therefore, while it is important to implement intervention programs to rectify IGD behaviors, it is also imperative to protect young adults from developing IGD through prevention strategies.

A previous study has examined the effects of the three basic psychological needs of autonomy, competence, and relatedness on problematic video game playing among Chinese young adults [5]. With more evidence surfacing that need dissatisfaction may be related to higher vulnerability for ill-being and psychopathology [25,26], the present study has incorporated the impact of need satisfaction and dissatisfaction for autonomy, competence, and relatedness in explaining IGD and flourishing as an indicator for eudaimonic well-being. The correlation results were generally in line with our hypotheses that psychological need dissatisfaction was positively correlated with IGD. On the other hand, the satisfaction of autonomy, competence, and relatedness needs was insignificantly or mildly related to IGD. When individuals’ psychological needs were dissatisfied, they would have a higher probability of using gaming as an escape from reality or some relief from a bad mood. In fact, these two reasons were found to be driving IGD among Chinese gamers [13].

Our hypotheses regarding psychological needs and flourishing were also largely supported by the correlational results, and flourishing was positively correlated to need satisfaction and negatively related to need dissatisfaction. Our results showed that once the basic psychological needs were satisfied, they would promote optimal human functioning. On the contrary, the dissatisfaction of psychological needs might hinder the actualization of one’s self and consequently undermine eudaimonic well-being.

Consistent with our prediction, flourishing was found to be negatively related to IGD. Continued and problematic online game playing would not only result in psychological distress [39] and lower sense of purpose in life [52], but also lead to an adverse effect on daily functioning [53,54]. In succession, it would affect eudaimonic well-being [55].

Based on the mediation analysis, only relatedness dissatisfaction was found to predict IGD significantly in our sample. An individual’s need for relatedness is suggested to be the most salient factor in explaining problematic gaming in Chinese young adults [5]. In many studies, individuals with need dissatisfaction for relatedness were reported to feel disconnected from their peers, and the feeling of isolation and loneliness was a risk factor for developing Internet-related addictions in Chinese emerging adults [5,56,57,58] who engaged in online activities like massively multiplayer online role-playing games (MMORPGs) as a source for new peer groups and social connection. Relatedness dissatisfaction gave rise to the feeling of loneliness and exerted a direct effect on developing symptoms related to IGD. Interpersonal connection is important for social animals like humans and serves as a protective factor for us. Although the underlying psychological mechanism/s between relatedness dissatisfaction and problematic game playing is yet to be determined, some research has suggested that teenagers and young adults, who are at the stage of establishing intimate relationships, may feel isolated if they fail and resort to playing online games [5,59]. Furthermore, some studies have found that relatedness dissatisfaction increased trait anger [59], and aggression was positively related to IGD [60]. A previous study has shown that higher levels of aggression would lead to goal-directed behaviors [61], as evidenced by gamers attempting to accomplish various goals in online games. Even though the process underlying relatedness dissatisfaction and problematic game playing remained unresolved, the effect of relatedness dissatisfaction on developing IGD and in turn lowering flourishing should not be neglected. Intervention programs are therefore recommended to increase social involvement of individuals and encourage participation in various activities. These initiatives may help to promote genuine relatedness with the community while reducing dissatisfaction [62].

## 5. Conclusions

Based on a large representative sample from the higher education vocational college in Mainland China, the present study enhanced our understanding of how different psychological need conditions, both satisfaction and dissatisfaction, are related to the development of IGD among Chinese young adults. The role of IGD in predicting flourishing under different conditions was also investigated. Our findings highlighted the indirect effect of relatedness dissatisfaction on flourishing through IGD. Yet, there are several research caveats that warrant attention. First, the present study adopted the cross-sectional design, hence it did not allow the attribution of causal relationship among the variables included herein. Second, the present study collected the data through a self-administered online survey and no clinical diagnosis for IGD was involved. Although we have already excluded the participants who failed to get at least four quality-check items and finished the survey in less than 15 minutes, to ensure the data quality and representativeness, a better probability sampling is recommended so as to generalize the results to the whole population. Moreover, the research did not look into the underlying mechanism between psychological needs and problematic game playing. Given the relatively high prevalence of IGD in Chinese young adults, a more comprehensive study covering a wider range of psychological constructs (e.g., the social motives of gaming and aggressiveness) and alternative measures of IGD in Chinese societies [63] is critical for a clear model in understanding the development of IGD. A multiwave longitudinal design in future studies may also elucidate both the protective and risk factors predicting the development and maintenance of IGD.

## Figures and Tables

**Table 1:** Summary of Means, Standard Deviations, and Intercorrelations for the Measures (*N* = 1200).

	*M (SD)*	2	3	4	5	6	7	8	9	10	11	12
Sex ^a^	—	−0.03	−0.08 **	−0.34 ***	−0.15 ***	−0.08 **	−0.04	−0.15 ***	−0.04	−0.02	−0.00	−0.06 *
Age	19.48 (1.21)	—	−0.19 ***	−0.13 ***	−0.05	0.07 *	−0.07 *	0.12 ***	−0.01	0.09 **	−0.05	0.13 ***
Hukou ^b^	—		—	0.19 ***	0.04	0.02	0.07 *	0.03	0.07 *	0.03	0.04	0.01
Daily gaming frequency	2.50 (1.92)			—	0.26 ***	0.06 *	0.06 *	0.01	0.08 **	−0.02	0.04	−0.06 *
Internet gaming disorder	1.14 (1.98)				0.84	−0.04	0.13 ***	−0.07 *	0.10 **	−0.03	0.19 ***	−0.14 ***
Autonomy satisfaction	3.40 (0.70)					0.66	0.15 ***	0.55 ***	0.29 ***	0.48 ***	0.05	0.43 ***
Autonomy dissatisfaction	2.84 (0.66)						0.42	0.11 ***	0.48 ***	0.16 ***	0.49 ***	−0.08 **
Competence satisfaction	3.45 (0.73)							0.80	0.23 ***	0.53 ***	−0.05	0.51 ***
Competence dissatisfaction	3.22 (0.73)								0.63	0.26 ***	0.46 ***	−0.00
Relatedness satisfaction	3.59 (0.80)									0.77	0.04	0.43 ***
Relatedness dissatisfaction	2.78 (0.79)										0.61	−0.22 ***
Flourishing	4.72 (0.97)											0.94

*Note.*^a^ Male = 1, Female = 2. ^b^ Rural = 1, Nonrural = 2. The reliability coefficients are found along the diagonal line. * p < 0.05. ** p < 0.01. *** p < 0.001.

**Table 2:** Model Coefficients for the Psychological Needs Mediation Analysis with Four Covariates (*N* = 1200).

					Outcome										
	Internet Gaming Disorder				Flourishing										
	Mediator (Model 1)				Total Effect (Model 2)				Direct Effect (Model 3)				Indirect Effect		
	*B*	*SE*	*t*		*B*	*SE*	*t*		*B*	*SE*	*t*		*B*	*SE*	BootCI
Sex ^a^	−0.36	0.13	−2.85	**	−0.03	0.05	−0.63		−0.04	0.05	−0.80		—	—	—
Age	−0.00	0.05	−0.06		0.04	0.02	1.98	*	0.04	0.02	1.98	*	—	—	—
Hukou ^b^	−0.10	0.13	−0.77		0.06	0.05	1.07		0.06	0.05	1.02		—	—	—
Daily gaming frequency	0.24	0.03	7.81	***	−0.03	0.01	−2.40	*	−0.02	0.01	−1.89		—	—	—
Autonomy satisfaction	−0.13	0.10	−1.38		0.29	0.04	7.33	***	0.29	0.04	7.26	***	0.003	0.003	[−0.002, 0.011]
Autonomy dissatisfaction	0.16	0.10	1.58		−0.08	0.04	−1.96	*	−0.08	0.04	−1.87		−0.004	0.003	[−0.011, 0.001]
Competence satisfaction	−0.16	0.10	−1.70		0.40	0.04	10.16	***	0.40	0.04	10.06	***	0.004	0.003	[−0.001, 0.012]
Competence dissatisfaction	0.02	0.09	0.23		−0.12	0.04	−3.06	**	−0.12	0.04	−3.05	**	−0.001	0.002	[−0.001, 0.004]
Relatedness satisfaction	0.02	0.08	0.28		0.24	0.03	6.91	***	0.24	0.03	6.94	***	−0.000	0.002	[−0.006, 0.004]
Relatedness dissatisfaction	0.37	0.08	4.38	***	−0.18	0.03	−5.27	***	−0.17	0.03	−4.98	***	−0.009	0.004	[−0.019, −0.002]
Internet gaming disorder	—	—	—		—	—	—		−0.02	0.01	−2.06	*	—	—	—
Constant	0.59	1.04	0.57		1.90	0.43	4.47	***	1.91	0.42	4.51	***	—	—	—
*R^2^*	0.11				0.37				0.38				—		
*F*	15.30 ***				72.70 ***				66.66 ***				—		

*Note.*^a^ Male = 1, Female = 2. ^b^ Rural = 1, Nonrural = 2. BootCI = Bootstrap 95% confidence interval for the indirect effect. * p < 0.05. ** p < 0.01. *** p < 0.001.
